# Study on multidimensional shrinkage spatial-temporal patterns and driving forces of cities in the Yellow River Basin

**DOI:** 10.1038/s41598-025-06292-3

**Published:** 2025-07-02

**Authors:** Guangrui Xing, Dongfeng Wang, Wanxin Cai

**Affiliations:** 1 College of Geographical Sciences, Faculty of Geographical Science and Engineering, Henan University, Zhengzhou, 450046 Henan China; 2 School of Culture and Tourism, Henan University, Kaifeng, 475001 China; 3 School of Civil and Hydraulic Engineering, Chongqing University of Science and Technology, Chongqing, 401331 China

**Keywords:** Urban shrinkage, Multidimensional, Spatial-temporal evolution, Random forest regression, YRB, Environmental social sciences, Sustainability

## Abstract

With economic globalization and the deepening process of industrialization and urbanization, China’s urban development has entered a vital transition stage. As one of the most influential rivers in China, the Yellow River Basin (YRB), with ecological protection and high-quality development as China’s national strategy, has not yet received sufficient attention for its urban shrinkage. Accordingly, this study constructs an evaluation index system for the shrinkage of cities in the YRB from four dimensions: population, economy, society and space. The entropy method and analytic hierarchy process are to determine the weights, the shrinkage model, the transfer matrix method and the exploratory spatial data analysis method are used to study the spatial-temporal evolution characteristics of 62 cities with data in the YRB. Random forest (RF) regression method was used to explore the influencing factors affecting the formation of urban shrinkage in the YRB and their influencing roles. The research results show that: (1) urban shrinkage in the YRB is characterized by spatial differentiation and shows a trend of drastic and concentrated development, with the shrinkage phenomenon becoming more and more significant; the degree of population shrinkage, economic shrinkage and social shrinkage is dominated by slight or moderate; the degree of space shrinkage and comprehensive shrinkage is dominated by high and heavy. (2) The reduction in the number of shrinking cities indicates a diminishing urban shrinkage across all dimensions, with a progressively increasing impact. (3) The accuracy of RF regression is high, and the main factors affecting the shrinkage of cities in the YRB account for 60.66%. An in-depth exploration of the characteristics of urban shrinkage and its development dynamics in the YRB from a multidimensional perspective will help to narrow the imbalance of urban development, promote high-quality development, and provide an essential reference to promote the progress of urban shrinkage research on a regional scale.

##  Introduction

Since the Second Industrial Revolution, many cities worldwide have experienced urbanization, which is reflected in their rapid population and economic growth^1^. As the level of global urbanization continues to deepen, there is a tendency for shrinking urban populations and economies of scale to spread to all corners of the world^2–6^. It is now widely recognized in the academic community that the root cause of urban shrinkage lies in the failure of the urban economy to shift effectively and promptly from traditional manufacturing to innovative high-tech industries and modern services, which has led to serious problems of unemployment and population loss^7^. Continued population decline has further weakened the economic strength of cities and exacerbated the Matthew and polarization effects among cities^8^. Urban shrinkage is one of the most important challenges in urban planning and regional development policies^9^. Therefore, it is necessary to identify the process of urban shrinkage and apply scientific urban policies to it^10^.

The distribution and causes of urban shrinkage have been fully explored in current international scholarly research^11^. Studies have shown that urban shrinkage is caused by the effects of continued depopulation, economic recession and oversupply of infrastructure^8^ageing^12^ resource depletion, and de-industrialization^11^ in addition to the effects of environmental change^13^. China, as a fast growing country in the world, is still experiencing rapid economic development and urbanization, and scholars generally agree that there are significant urban differences with Western countries^14^. In recent years, more and more studies have attempted to reveal the phenomenon of urban shrinkage in China’s cities, along with issues such as declining fertility rates, ageing populations^15^ and the “Lewis Tipping Point”^16^. Studies have shown that urban shrinkage has occurred not only in the northeastern and less developed regions in the central and western parts of the country, but also in the more economically developed regions of Beijing, Tianjin and Hebei, the Yangtze River Delta and the Pearl River Delta^17^. In response to this phenomenon, scholars using data from the fifth to seventh population censuses found that among the 261 prefectural-level cities in China for which data are available, urban shrinkage is characterized by a particular spatial differentiation, showing a drastic and concentrated development and a trend of increasing severity^18^. The problem of urban shrinkage is gradually attracting attention from various quarters, including society, government and many scholars.

There is no consensus in the academic community on the definition of urban shrinkage. The Shrinking Cities International Research Network (SCIRN) organization indicates that cities with a population size of 10,000 or more have experienced a population loss for at least two years, accompanied by a structural economic crisis^19^. Oswalt and Rieniets^20^. believe that urban shrinkage refers to a city whose population loss exceeds 10% of the total population, or whose annual population reduction rate exceeds 1%. Schilling and Logan^21^. consider shrinking cities to be older industrial cities that have lost more than 25% of their population in the last 40 years. On the contrary, some scholars argue that to identify them accurately shrinking cities is a comprehensive concept and should be a multidimensional process of change, which means combining population, economic, social^22^ and space dimensions^23^ and that a single indicator often fails to accurately identify them. Single indicators may introduce bias and fail to reveal the interdependence of the various elements of development^24^. To alleviate this problem, some scholars have used satellite remote sensing data, such as night lights, to assess the extent of urban shrinkage. This method has a high degree of objectivity and spatial resolution and provides complete time-series data that are not constrained by administrative boundaries^25,26^. However, as an indirect method of identifying urban shrinkage, nighttime lighting data is also subject to other issues, such as sensor resolution^27^. Accordingly, this study analyzes urban shrinkage in the YRB in four dimensions: demographic, economic, social and space. Previous research in the construction of urban shrinkage evaluation system mainly used the entropy method^28^ coefficient of variation method^29^ and other methods, subjective and objective weighting methods through the evaluation index system of the indicators of the subjective and objective weighting of the system, to reduce the bias, the use of its application is the primary trend of the future^30^. Spatial panel model^31^ multiple linear (ML) regression model^32^ partial least squares regression model^33^ and other methods are used to explore the influencing factors. However, scholars mostly use linear analysis model, which has many limitations and poor explanatory power in the face of complex nonlinear problems, while the nonlinear random forest (RF) model can overcome these shortcomings^34^.

**Fig. 1:**
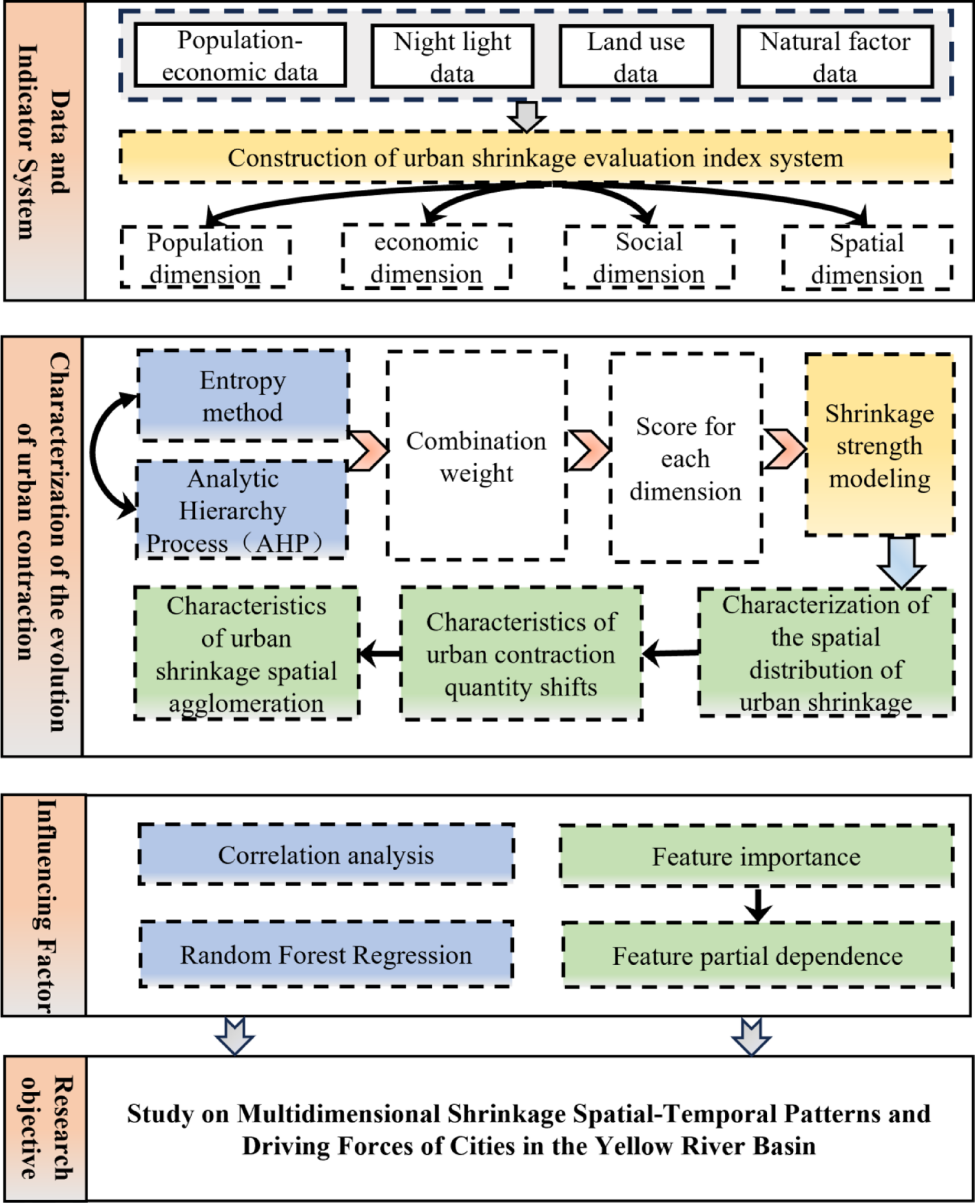
Research framework.

Some of the current research in China focuses on the shrinkage of resource-based and industrial cities, especially in the northeast, such as Fuxin and Fushun^35,36^. However, shrinkage is not limited to resource-based and industrial cities, and there are significant differences in population, economic, social, and policy factors in different regions or cities^37^. In 2019, ecological protection and high-quality development of the YRB have been elevated to China’s national strategy, and coordinated regional development is an essential support for deepening high-quality development. Therefore, studying the spatial pattern and driving force of urban shrinkage in the YRB is of great significance in accelerating the formation of a new pattern of coordinated regional development, promoting the high-quality development of the YRB region, and narrowing the economic development among cities in the YRB. The current research for the YRB has the following problems: (1) Lack of a multi-dimensional shrinkage evaluation system: most of the studies only analyze the population^38^ or population-economic dimensions^39^ or use nighttime lighting data to characterize the spatial dimension^40^which is incomplete in terms of dimensionality^41^ and insufficient to explain the spatial-temporal heterogeneity of shrinkage. (2) From the perspective of basin scale, existing studies have mainly investigated the phenomenon of urban contraction in developed basins, while the YRB, as a key area of China’s urban development, has been less studied accordingly. (3) From the perspective of research scale, the existing studies mostly take a certain industry or a certain type of city (resource-based city, old industrial city) as the research object, and the watershed research based on the unit of municipal area still needs to be strengthened. Therefore, this study constructs the evaluation system of urban shrinkage in the YRB from four dimensions: population, economic, social and space dimensions, and analyzes its spatial-temporal evolution characteristics, quantitative characteristics and spatial agglomeration characteristics by adopting shrinkage models, transfer matrices and exploratory spatial data analysis methods. The RF regression method was used to explore the factors affecting urban shrinkage in the YRB. It can provide scientific basis and decision support for the research and management of shrinking cities in the YRB (Fig. 1).

## Materials and methods

### Study area

**Fig. 2:**
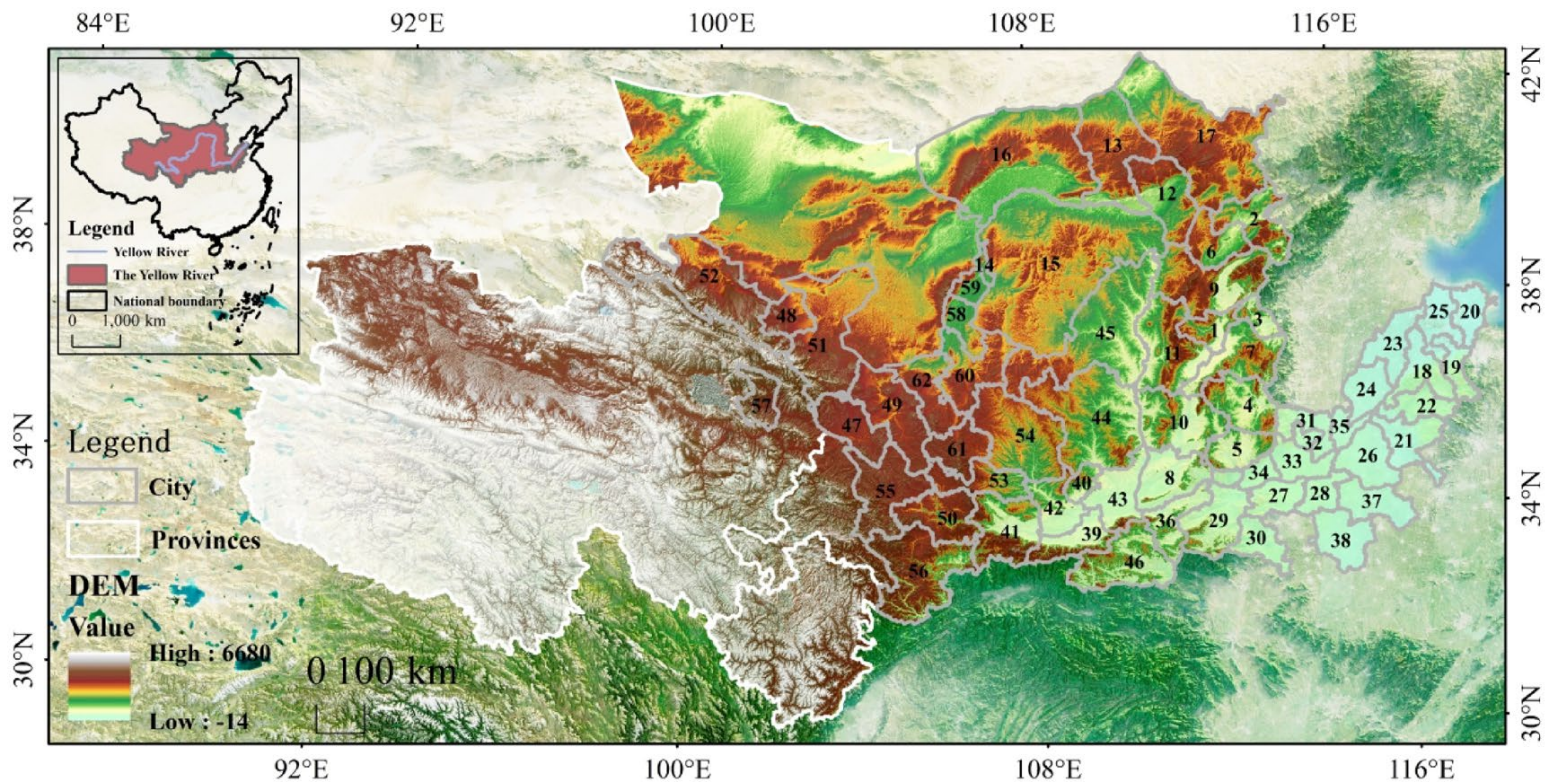
Study area. Note: 1-Taiyuan, 2-Datong, 3-Yangqian, 4-Changzhi, 5-Jincheng, 6-Shuozhou, 7-Jinzhong, 8-Yuncheng, 9-Xinzhou, 10-Linfen, 11-Luliang, 12-Huhhot, 13-Baotou, 14-Wuhai, 15-Ordos, 16-Bayannur, 17-Ulaanchab, 18-Jinan, 19-Zibo, 20-Dongying, 21-Jining, 22-Taian, 23-Dezhou, 24-Liaocheng, 25-Binzhou, 26-Heze, 27-zhengzhou, 28-Kaifeng, 29-Luoyang, 30-Pingdingshan, 31-Anyang, 32-Hebi, 33-Xinxiang, 34-Jiaozuo, 35-Puyang, 36-Sanmenxia, 37-Shangqiu, 38-Zhoukou, 39-Xi’an, 40-Tongchuan, 41-Baoji, 42-Xianyang, 43-Weinan, 44-Yan’an, 45-Yulin, 46-Shangluo, 47-Lanzhou, 48-Jinchang, 49-Baiyin, 50-Tianshui, 51-Wuwei, 52-Zhangye, 53-Pingliang, 54-Qingyang, 55-Dingxi, 56-Longnan, 57-Xining, 58-Yinchuan, 59-Shizuishan, 60-Wuzhong, 61-Guyuan, 62-Zhongwei. The base map used in the mapping is from the Standard Map Service System of the Ministry of Natural Resources of China (Map No. GS (2019) 1822, http://bzdt.ch.mnr.gov.cn/download.html), and the base map has not modified. Composed using ESRI ArcGIS 10.8 software. * This work is licensed under a Creative Commons Attribution (CC BY 4.0) license. ** ESRI, China.

The Yellow River originates from the Qinghai-Tibetan Plateau, with a total length of 5,464 km, and is the second largest river in China^42^. Complex social and economic factors and natural conditions add to the diversity of urban shrinkage. At the same time, the YRB has a large distribution of resource-based cities, and existing studies have shown that resource-based cities, as vital energy and heavy industry bases in China, ensure their high-quality economic and social development as an effective means to cope with social and ecological crises and promote sustainable development^43^. Taking the natural flow range of the YRB as the main body, considering the completeness of the administrative units and the correlation between the economic and social development of each province (region) and the YRB, and referring to the division of related research areas as well as the availability of data^44,45^ 62 cities with available data were selected as the objects of the study (Fig. 2). Since Laiwu was transferred to Jinan in 2019 and became the Laiwu District of Jinan, it was merged into Jinan for the survey. Pingdingshan, a designated resource-based city in China, is also studied as a research object. The YRB is divided into three regions: the upper reaches include Gansu Province, Inner Mongolia Autonomous Region, Ningxia Hui Autonomous Region, and Qinghai Province; the middle reaches include Shanxi and Shaanxi Provinces; and the lower reaches include Henan and Shandong Provinces.

### Data and pre-processing

The sources of vector and raster data and the data processing methods used in this study are shown in Table 1. The missing data of some cities were completed by linear interpolation method. All raster data were standardized to a resolution of 1 km and a coordinate system of GCS_WGS_1984 through the resampling tool, and the corresponding mean values were extracted by city through the tabular zoning statistics tool in ArcGIS 10.8.

**Table 1:** Data sources.

Data	Data type/resolution	Data period	Data sources	Data processing methods
Population, economic data	Raster	2006, 2011, 2016 and 2021	Statistical Yearbook of China’s Cities, Statistical Yearbook of China’s Regional Economy, Statistical Yearbook of China’s Urban and Rural Construction, and Statistical Yearbook of Provinces and Municipalities in the YRB.	Some of the missing data were filled in using linear interpolation.
Nighttime Lighting Data	Grid /1km	2005–2020	https://data.tpdc.ac.cn/	–
Annual average temperature data	Grid /1km	2005–2020	https://data.tpdc.ac.cn/	Processing the month-by-month mean temperature raster through the Raster Calculator tool in ArcGIS 10.8 to obtain the year-by-year mean temperature raster.
Annual average rainfall data	Grid /1km	2005–2020	https://data.tpdc.ac.cn/	Monthly average rainfall rasters were processed by the Raster Calculator tool in ArcGIS 10.8 to obtain yearly average rainfall rasters.
PM2.5 concentration data	Grid /1km	2005–2020	https://data.tpdc.ac.cn/	–
DEM	Grid /90m	–	http://www.resdc.cn	DEM data of the study area was obtained by mosaicking nine provinces (districts) in ArcGIS10.8
Land use data	Grid /1km	2005–2020	http://www.resdc.cn	Through ArcGIS10.8 reclassification into 6 categories of arable land, forest land, grassland, waters, construction land and unutilized land, and extracted construction land
NDVI data	Grid /1km	2005–2020	http://www.resdc.cn	–

### Research methods

#### Multidimensional urban shrinkage evaluation system

The essence of urban shrinkage is due to the decline of the attractiveness of the city itself and the outflow of population, which leads to the market behavior of repositioning of various development elements that affect urban development^18^. A single evaluation system is often insufficient to characterize the phenomenon of urban shrinkage fully, and too many evaluation indicators can instead obscure the core features of urban shrinkage^46^. Accordingly, this study is based on the essence of urban shrinkage, synthesizes the existing relevant research results^18^ and fully considers the characteristics of the population, economy, industrial structure, infrastructure, and other factors of cities in the YRB, as well as the availability and typicality of data, to construct an urban shrinkage evaluation index system from four dimensions: population, economy, society, and space (Table 2). In the population dimension, the decline in population is the most obvious manifestation of urban shrinkage, while the increase in population helps to effectively curb this trend and slow down the process of urban shrinkage. Population per unit of employment can reflect the employment situation in a city. When the number of persons per unit of employment is low, it means that there are limited employment opportunities, which will lead to an increase in the loss of urban population in order to make a living^47^. In the economic dimension, as the core factor driving the rapid development of cities, the strength of the economy has a direct impact on the growth potential of cities. The stronger the economy, the greater the city’s growth potential, which in turn makes it relatively less likely to face the risk of shrinkage. GDP reflects the city’s speed of development, and as GDP growth slows down, the city is more likely to experience shrinkage. GDP per capita reflects a city’s level of economic development, and the higher the level of economic development, the stronger the ability to withstand shrinkage. Local general fiscal contraction reflects the stability of economic development; the higher the local revenue, the greater the stability of economic development and the less likely shrinkage will occur. The average wage of employees reflects the income level of residents; the higher the average wage of employees, the stronger the city’s absorptive capacity for population and the lower the risk of shrinkage^48,50^. In the social dimension, The core objective of urban development is to provide residents with comprehensive public services, a good living environment, convenient transportation and quality educational resources, so as to have the core competitiveness to attract and retain talents. It has been proven that if a city fails to ensure quality living and education conditions for its residents, it may accelerate the decline of urban functions and population loss, and the possibility of urban shrinkage is high. Total retail sales of consumer goods reflects residents’ consumption. The higher the proportion of total retail sales of consumer goods, the greater the demand for consumption and the stronger the self-regulation ability of the market; the less likely that shrinkage will occur. The number of medical beds per capita reflects the level of medical protection in the city; the more adequate the medical resources, the less residents will worry about the shortage of medical resources in the event of a major public health event; the lower the probability of urban shrinkage. Road area per capita reflects the city’s transportation convenience. The more convenient the city’s transportation is, the more attractive the city is to talents. The number of primary and secondary school students reflects the city’s future development potential; the more primary and secondary school students there are, the greater the city’s development potential^51–53^. In the space dimension, Urban space is the driving force and important engine of urban development, and urban space can reduce the likelihood of shrinkage. The greater the average nighttime light intensity value, the more frequent the human activities in the area, and the lower the probability of urban shrinkage. Empty city index is the ratio of resident population to built-up area. The smaller the empty city index, the lower the probability of urban shrinkage^23,54^.

**Table 2:** Evaluation index system and weights of urban shrinkage in the YRB.

dimension	Sub-indicators	Unit	Indicators	Objective weights	Subjective weights	Combined weights
population	Resident population	10000people	Size of population	0.060	0.131	0.097
	Unit employed population	10000people	Population employment	0.089	0.261	0.167
Economy	GDP	100 million yuan	Economic strength	0.114	0.024	0.057
	GDP per capita	yuan	State of economic development	0.071	0.089	0.087
	Local fiscal revenue	100 million yuan	Economic strength	0.149	0.055	0.099
	Average wage of employees	yuan	Residential income	0.063	0.153	0.107
Social	Total retail sales of consumer goods	100 million yuan	Consumption status of the population	0.147	0.064	0.107
	Number of medical beds per capita	sheet	Status of medical protection	0.036	0.015	0.026
	Urban road area per capita	m^2^	State of infrastructure	0.046	0.024	0.036
	Number of elementary and middle school students in school	10000people	Educational status	0.064	0.041	0.056
Space	Average brightness of night light	–	Human activity	0.067	0.096	0.088
	Empty city index	–	Living environment	0.093	0.048	0.073

#### Combined weights solution

To avoid the influence of the difference in the scale of each indicator on the calculation results, the indicators are standardized. Different standardization treatments are used for positive and negative indicators, and the calculation formula is as follows^55^:

$$\begin{aligned} & {\text{For positive indicators: }}x_{{ij}}^{\prime }=\frac{{{X_{ij}} - \hbox{min} \left( {{X_{ij}}} \right)}}{{\hbox{max} \left( {{X_{ij}}} \right) - \hbox{min} \left( {{X_{ij}}} \right)}} \\ & {\text{For negative indicators: }}x_{{ij}}^{\prime }=\frac{{\hbox{max} \left( {{X_{ij}}} \right) - {X_{ij}}}}{{\hbox{max} \left( {{X_{ij}}} \right) - \hbox{min} \left( {{X_{ij}}} \right)}} \\ \end{aligned}$$

where *i* is the indicator number; *X*_*ij*_ is the actual calculated value; max*X*_*i*_ and min*X*_*i*_ are the maximum and minimum values of the *i* indicator respectively.

To reduce the impact of subjective factors and the degree of data dispersion on the weights. In this study, through the entropy value method and hierarchical analysis method, objective weight *W*_*1*_ and subjective weight *W*_*2*_ are calculated, and subjective and objective weights are synthesized with the help of the principle of minimizing information entropy. This method can effectively reduce the bias in the process of studying urban shrinkage, and finally get the comprehensive weight *W*_*i*_, the specific calculation formula is^56^:

$${W_i}=\frac{{\sqrt {{W_{i1}} \times {W_{i2}}} }}{{\sum\nolimits_{1}^{n} {\sqrt {{W_{i1}} \times {W_{i2}}} } }}$$

where *W*_*i1*_ and *W*_*i2*_ are the weights calculated by entropy method and analytic hierarchy process respectively.

#### Shrinkage model

By calculating the intensity of urban shrinkage, it is conducive to the spatial and temporal evolution characteristics of urban shrinkage in the YRB, and to grasp the dynamics and laws of urban shrinkage in the YRB. The calculation formula is as follows^23^:

$$CS_{{({t_1},{t_2})}}^{k}=\frac{{{X_{{t_2}}} - {X_{{t_1}}}}}{{{X_{{t_1}}}}}$$

Where CS is the shrinkage intensity of city k during the period, and ${X_{{t_1}}}$ and ${X_{{t_2}}}$ are the values of indicator *i* in years *t*_*1*_ and *t*_*2*_, respectively.

#### Transfer matrix

This study based on the classification of contraction in cities, using the transfer matrix of land use type of method, different dimensions of each period of the Yellow River city urban development type distribution is analyzed. Through the transfer matrix method, the quantity distribution and transfer direction of urban shrinkage types is discussed, which can show the dynamic change process of urban shrinkage directly^29^.

#### Exploratory Spatial data analysis methods (ESDA)

The exploratory spatial data analysis method explores the spatial correlation between the distribution of spatial variables and adjacent variables through statistical description and visualization of the distribution pattern of the research objects. It reveals the interaction mechanism of the research objects in the spatial pattern^57^.

1. Global spatial autocorrelation
  Global spatial autocorrelation is an indicator that describes the degree of association among all spatial units in the study area, and the method is used in this study to reveal the spatial agglomeration characteristics of urban shrinkage in the YRB. The formula is as follows:
  $$I=\frac{{\sum\limits_{{i=1}}^{n} {\sum\limits_{{j \ne i}}^{n} {{\omega _{ij}}\left( {{x_i} - \bar {x}} \right)\left( {{x_i} - \bar {x}} \right)} } }}{{\left( {\sum\limits_{{i=1}}^{n} {\sum\limits_{{j \ne i}}^{n} {{\omega _{ij}}} } } \right)\sum\limits_{{i=1}}^{n} {{{\left( {{x_i} - \bar {x}} \right)}^2}} }}$$
  where *x*_*i*_ and *x*_*j*_ are the comprehensive shrinkage indices for regions *i* and *j*; *n* is the total number of cities; ${\omega _{ij}}$ is the regional spatial weight matrix; $\bar {x}$ is the average value of *x*; *I* is the spatial autocorrelation index. When *I* > 0, it indicates a positive correlation; when *I* < 0, it indicates a negative correlation; and when *I* = 0, it means that all attributes are randomly distributed.
2. Local spatial autocorrelation.
  Local spatial autocorrelation can reveal specific spatial agglomeration areas and explore the identification of spatial clustering patterns to further enhance the level of knowledge about urban shrinkage from a spatial perspective. The formula is as follows:
  $${I_i}=\sum\limits_{{j=1}}^{n} {{\omega _{ij}}} {x_j}/\sum\limits_{{i=1}}^{n} {{x_i}}$$
  Based on *I*_*i*_, four clustering patterns can be formed, and the resulting Local Indicators of Spatial Association (LISA) distribution maps can show the clustering characteristics. The clustering patterns can be categorized into H-H clustering, L-L clustering, L-H clustering and H-L clustering.

#### Methodology for analyzing impact factors

##### Selection of influencing factors

This study takes five years as a time unit, and the social and economic factors are selected using the starting year data to fully account the lag of the urban shrinkage phenomenon. The following indicators were selected: (1) Number of people is the most direct manifestation of urban shrinkage and a key criterion for judging whether a city is shrinking or not. Cities must have enough labor force to develop, but China’s structure of Population is undergoing a historic change, and the reduction of labor force seriously affects the development of society, economy and other aspects^58^. (2) The natural population growth rate reflects the future growth potential of the city. A long-term low or negative growth rate will result in a shortage of young labor, exacerbate the problem of population aging, and accelerate the process of urban shrinkage^59^. (3) Population agglomeration. Excessively high population density may trigger the deterioration of the living environment and resource overload, while excessively low population density leads to a decline in the utilization rate of infrastructure and the escalation of public service costs. Therefore, the spatial scale of population agglomeration is conducive to the formation of a certain agglomeration effect in the region, and generate a certain economy of scale to promote urban development, and vice versa is not conducive to urban development and may lead to urban shrinkage^60^. (4) The size of the unemployed population is directly related to the economic vitality of the city. A high unemployment rate often means that the region is unable to provide a better standard of living and more employment opportunities, triggering an exodus of labor and a contraction of consumer demand, which in turn leads to urban shrinkage^9^. (5) Industrialization and service level. The level of industrialization reflects the resilience of the city’s economic structure, and the high proportion of traditional industries is susceptible to the impact of resource constraints and fluctuations in the industrial cycle, and if it fails to carry out industrial upgrading in a timely manner, it may lead to a wave of enterprise closures and job losses, resulting in structural shrinkage. The service industry is the main source of employment absorption in cities. The backwardness of the service industry will limit the employment of the labor force, exacerbate the exodus of the young population, and lead to urban shrinkage^10,22^. (6) The proportion of science and technology education expenditure. The proportion of science and technology investment reflects the city’s innovation capacity. Insufficient investment in science and technology will lead to the loss of development opportunities in the regional competition, leading to the development of the city lagging behind, which in turn triggers urban shrinkage. Education investment affects the future development of the city. Too little investment in education will affect the quality of future talent, thus hindering the transformation and upgrading of urban industries, and at the same time, people will migrate in search of quality education, accelerating urban shrinkage^47,61^. (7) Industrial output value reflects the vitality of the city’s industrial development. The decline of industrial output value means the decline of pillar industries, which directly leads to the reduction of jobs, and also leads to the relocation of industry-related enterprises, triggering urban shrinkage^62^. (8) Fixed asset investment reflects the city’s infrastructure development. Reduced investment will lead to aging infrastructure and fewer industry-related facilities, reducing the attractiveness of the city; inducing urban shrinkage^63^. (9) Ecological quality. Too low a green coverage in built-up areas will exacerbate the heat island effect and ecological degradation, and reduce the livability of the city; whereas a high coverage can attract population agglomeration and inhibit the shrinkage trend by enhancing ecological service functions. Urban air quality greatly influences people’s decision-making about where to live, and is an important consideration when choosing where to live. Good urban ambient air quality tends to attract people to live and work in cities. On the contrary, poor air quality poses a potential threat to the health of residents, which will drive a portion of the population, especially young people and high-income groups, to choose to move away from more polluted cities. A continuous decrease in the NDVI value implies land degradation or shrinkage of ecological space, which will lead to a poor urban environment and accelerate population exodus, which will then lead to shrinkage of the city^37,64,65^. (10) Slope conditions and elevation conditions. It is an important natural factor that affects the construction and development of the city. Areas with greater relief or higher elevation usually face higher construction costs and technical difficulties, leading to more difficult infrastructure construction and urban expansion. These areas are often unsuitable for large-scale urban development because complex topographic conditions can limit population settlement and productive activities^66^. (11) Temperature conditions and rainfall conditions. Climate comfort is a hidden push for population migration. Extreme weather conditions increase the cost of living and health risks, driving population migration to areas suitable for population survival, which in turn affects urban shrinkage^67^ (Table 3).

**Table 3:** Influencing factors of urban shrinkage in the YRB.

Variable name	Variable	Variable declaration
Total population at year end	X_1_	Total population at the end of the starting year of the unit period
Natural population growth rate	X_2_	Natural population growth rate per unit period, starting year
Population density	X_3_	Starting annual population density per unit period
Urban registered unemployed population	X_4_	Registered unemployed population in urban areas in the starting year of the unit period
Industrialization level	X_5_	Starting year of unit period (added value of secondary and tertiary industries/GDP of region)
Service level	X_6_	Starting year of unit period (value added of tertiary industry/value added of secondary and tertiary industries)
Science as a proportion of fiscal expenditure	X_7_	Starting year of unit period (science expenditure/financial expenditure)
Education as a proportion of fiscal expenditure	X_8_	Starting year of unit period (education expenditure/financial expenditure)
Gross industrial output value above scale	X_9_	Gross value of industrial output above designated size for the beginning of the unit period
investment in fixed assets	X_10_	Investment in fixed assets in the starting year of the unit period
Greening coverage in built-up areas	X_11_	Greening coverage of built-up areas in the starting year of the unit period
PM2.5 concentration	X_12_	Starting annual PM2.5 concentration per unit period (raster resolution of 1 km)
Average altitude	X_13_	Average elevation (raster resolution of 90 m)
Average slope	X_14_	Average slope (raster resolution of 90 m)
Terrain relief	X_15_	Average terrain relief (raster resolution of 90 m)
Average annual temperature	X_16_	Average air temperature (raster resolution of 1 km)
Average annual rainfall	X_17_	Average rainfall (raster resolution of 1 km)
NDVI	X_18_	NDVI per unit period start year (raster resolution 1 km)

##### Regression model and validation

The influencing factors of the urban shrinkage phenomenon in the YRB were explored using each influencing factor in Table 3 as the independent variable and the calculated shrinkage intensity as the dependent variable. Linear regression models are often used to analyze the factors influencing urban shrinkage in previous studies. The RF regression model used in this study is more tolerant to noise, less prone to overfittin^68–70^and can better identify the influencing factors of urban shrinkage in the YRB. Calling the sklearn module for RF regression on the Python platform. After several debugging sessions, the parameters n_estimators = 800, criterion = MSE, and the rest of the parameters were selected by default. The study used the coefficient of determination R^2^ and mean square error (MSE) as indicators for evaluating the accuracy of the regression model. The coefficient of determination R^2^ is the degree of fit of the fitted value to the observed value after regression, and its value range is [0,1]; the closer R^2^ is to 1 indicates better fit; the MSE is the average of the sum of squares of the residuals, the smaller the MSE, the smaller the error.

## Results and analysis

### Characteristics of the spatial distribution of urban shrinkage in the YRB

Using the multi-indicator comprehensive evaluation method to analyze each of the four dimensions that make up urban shrinkage, as well as the comprehensive dimension, existing studies have shown that slow growth often means a decline in the city’s attractiveness and leads to a relative shrinkage of the city^71^. Therefore, this study uses a relative shrinkage approach to assess the shrinking state of cities. When the CS of a city is higher than the average level of the YRB, it is defined as no shrinkage. The natural breakpoint method is used to grade the shrinkage of the remaining cities, which can be divided into slight shrinkage, moderate shrinkage, severe shrinkage, extreme shrinkage and no shrinkage.

**Fig. 3:**
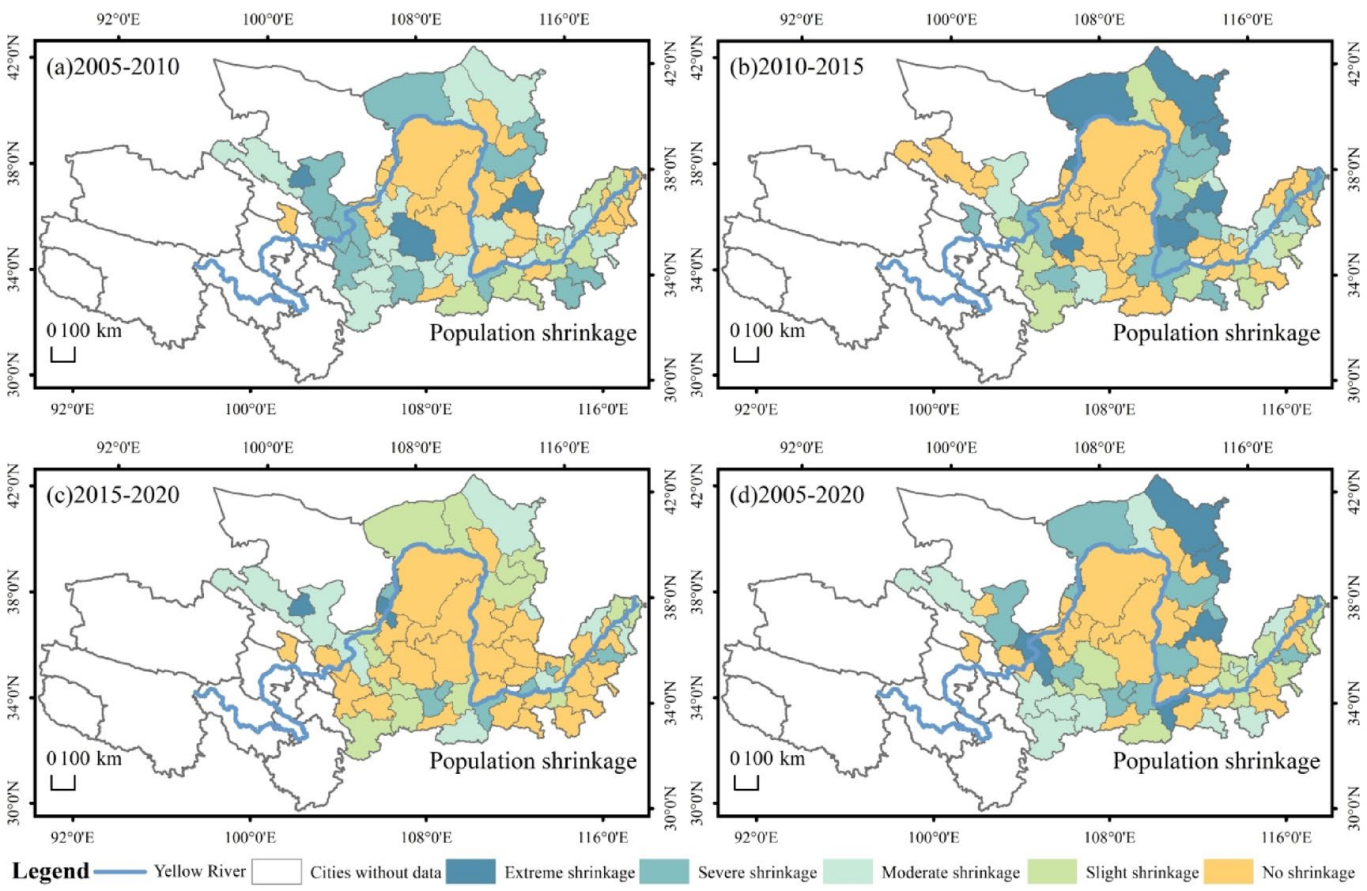
Spatial distribution of shrinking population dimensions in the YRB.

The spatial distribution of urban population shrinkage in the YRB can be seen in Fig. 3. The number of cities in which shrinkage occurred in 2005–2010 was 40, the number of cities in which shrinkage occurred in 2010–2015 was 38, and the number of cities in which shrinkage occurred in 2015–2020 was 33, with the distribution of cities shrinking in the three periods characterized by dispersion and aggregation. In 2005–2010, 2010–2015 and 2015–2020, 28, 17 and 16 cities, representing 70%, 45% and 48% of all cities experiencing population shrinkage, respectively, will experience slight and moderate population shrinkage. In contrast, there has been a gradual increase in the proportion of severe and extreme population shrinkage in cities (Fig. 3a, b and c). In 2005–2020, the cities with severe and extreme shrinkage were mainly in the upper regions; the distribution of shrinking cities was scattered, and shrinking cities formed multiple contiguous agglomeration areas (Fig. 3d). With time, the population from the western region slowly to the central and eastern areas of urban concentration, mainly due to these regions than the western region of infrastructure and supporting facilities, developed education and medical level, more jobs, attracting a large number of employment inflow, ultimately leading to the central and eastern regions gathered a large number of inflow of population, the western region population in further decline. It can also be foreseen that due to China’s population policies, such as family planning in the early period, the disappearance of the population dividend, and the impact of labour migration and capital outflow due to the imbalance of development between regions, the number of cities with population contraction in the YRB will further increase in the future, and the extent of the contraction will be further aggravated, and the scope of distribution will be further enlarged.

**Fig. 4:**
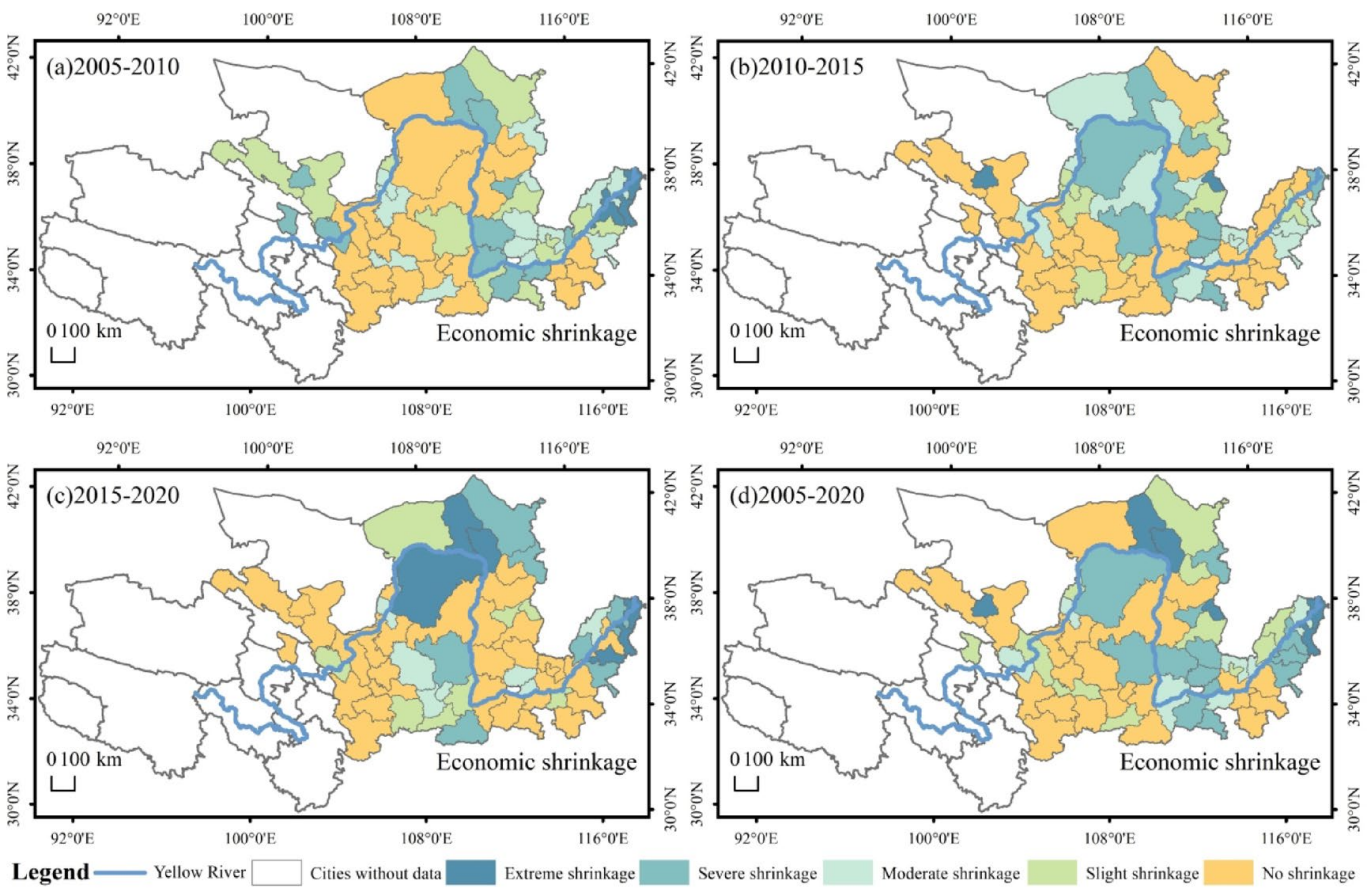
Spatial distribution of economic dimension shrinkage in the YRB.

The spatial distribution of economic shrinkage of cities in the YRB can be seen (Fig. 4). The number of cities experiencing economic shrinkage from 2005 to 2010 was 63%, mainly in the more economically developed middle and lower regions. Severe and extreme shrinkage cities accounted for 33% of the cities experiencing shrinkage, with extreme shrinkage cities occurring in the coastal region (Fig. 4a). In 2010–2015, 53% of the cities experienced economic shrinkage, mainly in the central region, and economic shrinkage cities showing a clear trend of movement towards the middle of the region. Cities experiencing severe and extreme shrinkage accounted for 48% of the overall economic shrinkage cities, with fewer cities experiencing economic shrinkage in this period than in the previous one, but overall no large-scale economic recession occurred. This was made possible by China’s “4 trillion” support program in 2009 in response to the financial crisis, which mitigated the impact of the financial crisis on the cities in the YRB during this period (Fig. 4b). In 2015–2020, the YRB is gradually deepened by the impact of the national financial crisis, and three agglomerations are formed in the urban shrinkage of the economy. Although the amount of cities experiencing economic shrinkage has decreased, severe and extreme shrinkage accounted for 48% of the total number of cities experiencing economic shrinkage. Cities experiencing shrinkage are mainly located in Shandong, Shaanxi and more economically developed areas, which means that the ability to withstand financial risks is weaker, and even if there is a shrinkage of the urban economy, the shrinkage is less severe. Cities with severe shrinkage are dispersed between the upper and lower regions, which may be due to differences in the level of economic development between regions and different development dynamics (Fig. 4c). Cities experiencing economic shrinkage in 2005–2020 are mainly in the lower and middle areas, with increasing levels of shrinkage (Fig. 4d). Municipal economic shrinkage is more concentrated in the lower areas and more dispersed in other municipalities. In the middle reaches of the Yellow River, predominantly resource-dependent cities experienced more significant shrinkage. From a spatial distribution perspective, the shrinking cities were sequentially distributed across administrative divisions. The possible reasons are that the ecological environment of the Yellow River has been gradually emphasized, and some resource-based cities have experienced a gradual deepening of economic shrinkage due to untimely transformation.

**Fig. 5:**
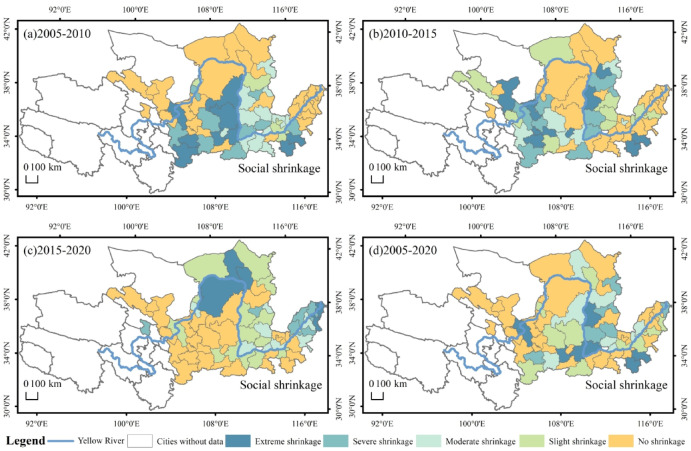
Spatial distribution of shrinkage of social dimensions in the YRB.

The distributional characteristics of urban social shrinkage in the YRB are shown in Fig. 5. In 2005–2010, the shrinking cities were 58%, mainly in the middle reaches of the Yellow River. (Fig. 5a). In 2010–2015, 55% of the cities experienced social shrinkage, a decrease from the previous period, mainly concentrated in Gansu and Ningxia regions, where the shrinkage was more severe, while the remaining cities with social shrinkage were sporadically distributed in lower regions (Fig. 5b). In2015-2020, the social shrinkage of cities showed a trend of moving to the middle and lower reaches, mainly concentrated in Shanxi and Shandong provinces, and the social shrinkage of Shandong cities was more serious than that of cities in other regions, and the other cities with social shrinkage were scattered (Fig. 5c). By comparing the cities of social shrinkage between 2010 and 2015 and 2015–2020, it can be found that there were almost no cities of social shrinkage between 2010 and 2015 in Henan and Shandong provinces, but there were a large number of cities of social shrinkage in 2015–2020, especially in Shandong, where 8 out of 9 cities showed shrinkage. In 2005–2020, urban shrinkage will occur mainly in the middle and lower regions, with resource-based urban shrinkage being more severe in the middle regions. (Fig. 5d). It can be foreseen that if the cities in the YRB fail to carry out technological upgrading and transformation in time in the future, especially the weakening of the gravitational attraction of traditional industries to talents, capitals and other factors in resource cities, the phenomenon of social shrinkage of the cities will be further deepened in the future.

**Fig. 6:**
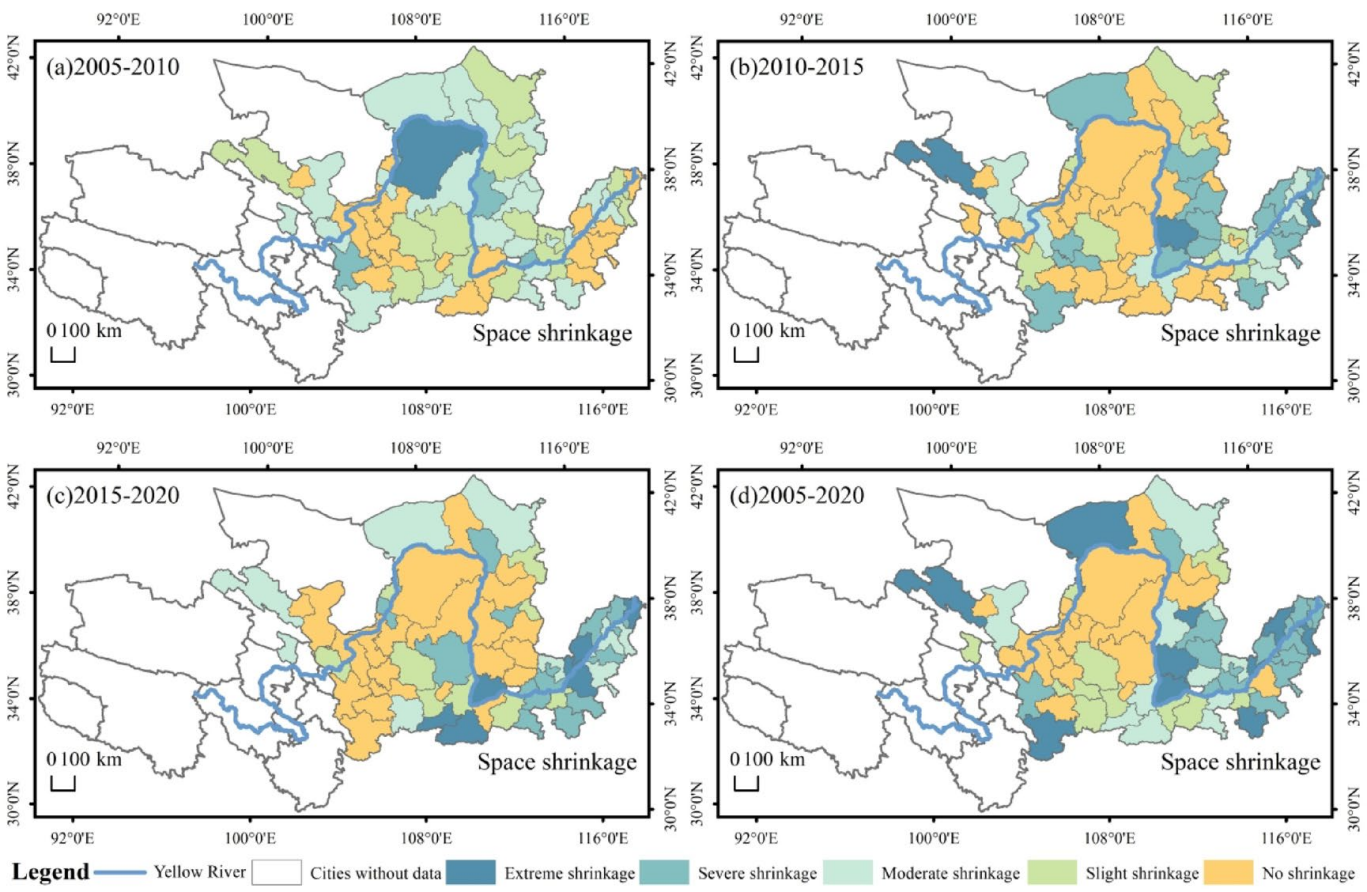
Spatial distribution of space dimension shrinkage in the YRB.

The spatial distribution of space dimensions in the YRB is shown in Fig. 6. In 2005–2010, the cities with space shrinkage mainly in the middle and upper reaches, accounting for 71%. The cities experiencing space shrinkage were primarily concentrated in Inner Mongolia, Shanxi, and Shaanxi, while other instances of space shrinkage were dispersed across Shandong and Gansu. (Fig. 6a). In 2010–2015, the cities with spatial shrinkage were mainly in the middle and lower reaches of the Yellow River, and the degree of shrinkage gradually deepened, with cities with severe and extreme shrinkage accounted for 71% of the cities with space shrinkage in this period (Fig. 6b). In 2015–2020, 64% of the cities experience shrinkage in the space dimension, mainly in the middle and lower reaches, with a few cities also shrinking in Inner Mongolia and Gansu. The degree of space shrinkage deepened further in the lower reaches of the region, with 67% of the cities in severe and extreme shrinkage (Fig. 6c). In 2005–2020, 74% of the cities undergoing space shrinkage. The cities with heavier shrinkage are mainly in Shandong, Henan and Shanxi regions accounting for 80% of the cities undergoing space shrinkage. Most of the cities experiencing space shrinkage are resource-based cities. The likely reason is that resource-based cities have experienced a gradual worsening of the phenomenon of urban shrinkage as a result of untimely industrial restructuring, which has led to a shift in the mode of production based on resource exploitation, resulting in a massive exodus of the population (Fig. 6d).

**Fig. 7:**
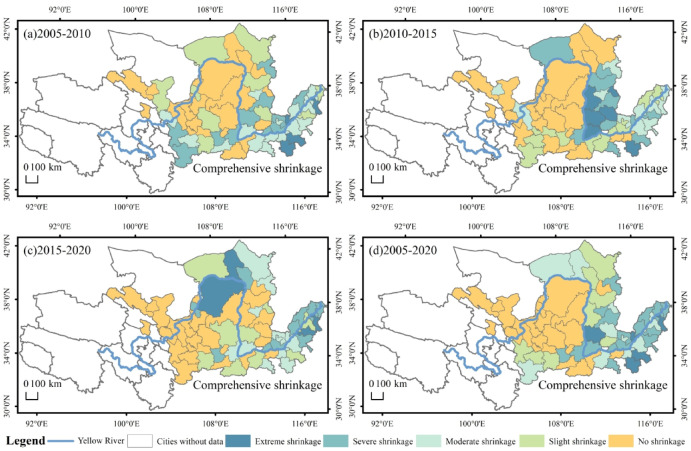
Spatial distribution of comprehensive dimensional shrinkage in the YRB.

The spatial distribution of the comprehensive dimensions in the YRB is shown in Fig. 7. In 2005–2010, 65% of the cities experienced comprehensive shrinkage, of which 30% were severe and extreme shrinkage cities, located in Gansu, Henan and Shandong (Fig. 7a). In 2010–2015, cities experienced shrinkage in the comprehensive dimension were mainly clustered in the lower and middle regions, with 63% of the cities shrinking, of which severe and extreme shrinkage accounted for 44% of the shrinking cities. Especially in Shanxi, Henan, Shandong region formed a large shrinkage area, shrinkage of the heavier cities clustered in the Shanxi region, the rest of the shrinkage of the city scattered distribution in Gansu, Shaanxi, Inner Mongolia region (Fig. 7b). In 2015–2020, 58% of cities experienced shrinkage in the comprehensive dimension, with 44% of cities experiencing severe and extreme shrinkage. A contiguous agglomeration of shrinkage has formed in the lower reaches of the region. The distribution of extreme shrinkage cities is relatively decentralized (Fig. 7c). The comprehensive shrinkage in 2005–2020 is 65%, and the heavier shrinkage is also predominantly in the lower reaches of the city (Fig. 7d). By comparing the three periods with the 2005–2020 comprehensive dimension shrinkage cities can be found, the spatial distribution of the change is not large, but the severity of the shrinkage gradually deepened.

### Characteristics of shrinkage quantity shift of cities in the YRB

**Fig. 8:**
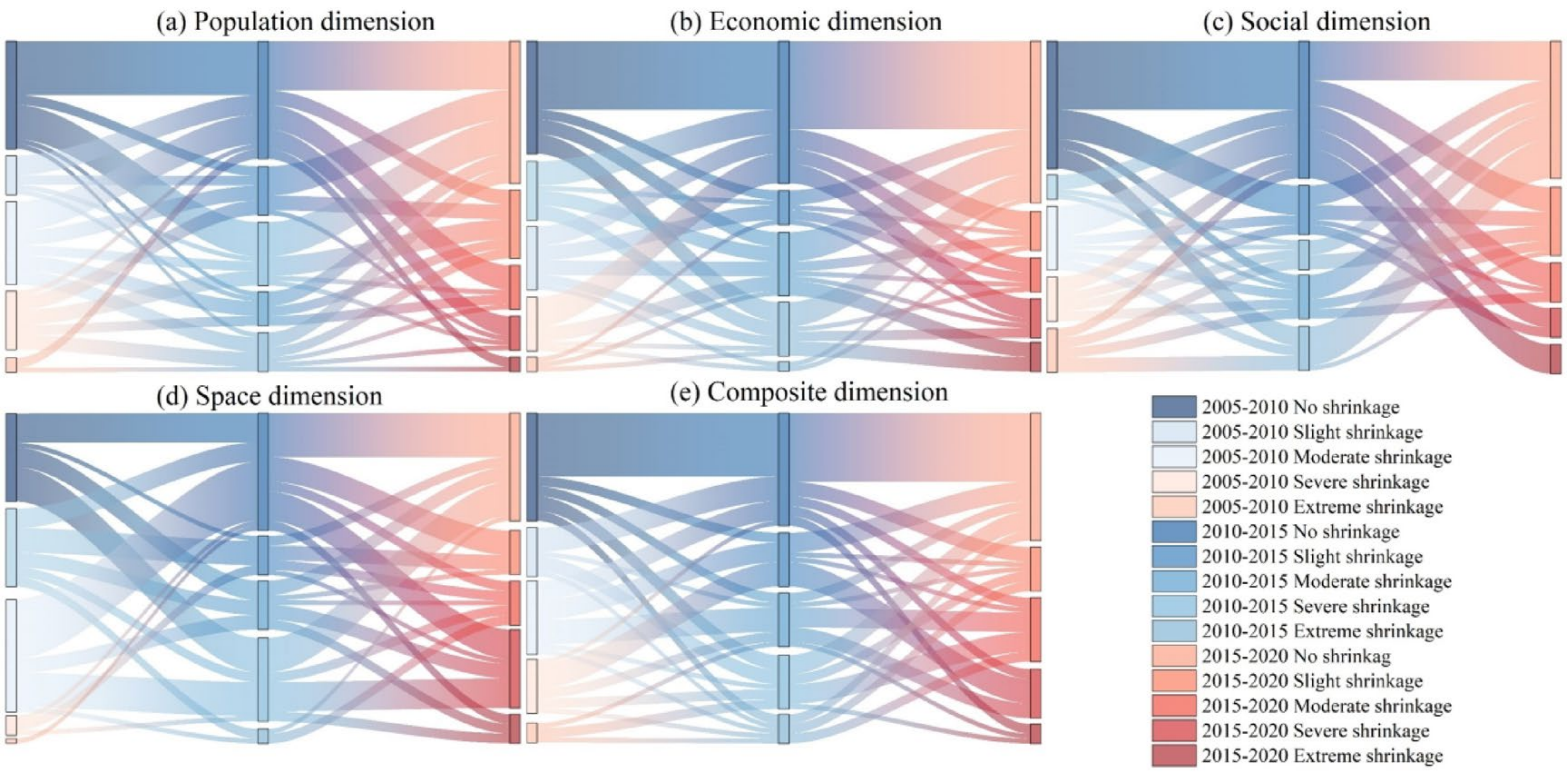
Transfer matrix of dimensions in the YRB.

The number of cities with different dimensions of growth and shrinkage types can be seen in Fig. 8, and the percentage of cities in the unshrinkage type is higher than that of other cities in the same dimension, indicating that growth is still the primary trend of urban development in the YRB. With time, the demand for transformation and upgrading of urban industrial structure and the contradiction between ecological environmental protection and urban development have intensified. The number of urban growth has declined, and urban development has become increasingly slow. On the population dimension, there were 21 cities with increasing shrinkage from 2005 to 2010 to 2010–2015, 21 cities with decreasing shrinkage (including a shift to a growth type), nine cities with no change in shrinkage, and 11 cities that were always growing. The number of cities with increased shrinkage from 2010 to 2015 to 2015–2020 rose to 22, with ten cities constantly growing, and 23 cities with diminished shrinkage, an increase of 9%. Overall, the number of cities with weakened shrinkage is roughly the same as the number of cities with increased shrinkage. This may be due to the fact that the cities with increased shrinkage are lagging behind in terms of development and are losing population as a result of reduced employment opportunities, whereas the cities with decreased shrinkage in the demographic dimension may be due to a “siphoning effect”, which leads to a decrease in the degree of shrinkage of the city (Fig. 8a). On the economic dimension, there were 19 cities experienced an increased level of shrinkage from 2005 to 2010 to 2010–2015, while five cities showed no change in shrinkage level, accounting for 31% and 8% respectively. Additionally, there were 24 cities where the level of shrinkage decreased, accounting for 39%. Overall, the number of cities experiencing economic contraction is gradually decreasing, possibly because some cities have raised the income level of their residents by promoting the development of high-technology industries, so the degree of economic contraction has weakened, but the cities with increased economic contraction may be due to the industrial transformation of the city and the decline in city revenues, which has led to slow economic development, and in turn to an increased degree of contraction (Fig. 8b). On the social dimension, there are 18 cities where the shrinkage worsened from 2005 to 2010 to 2010–2015, accounting for 29% of the total, six cities where the shrinkage remained unchanged, and 24 cities where the shrinkage weakened. From 2010 to 2015 to 2015–2020, the number of cities with increased shrinkage increased by five from the previous period, with the main direction of shift being from cities with low shrinkage to cities with high shrinkage. There were three cities where the degree of shrinkage remained unchanged. Four more cities where the degree of shrinkage weakened from the previous period. Overall, the growing number of cities with increased shrinkage may be attributed to increased social problems and deteriorating social conditions due to the city’s spending power, weak educational resources and fewer public facilities, which further exacerbate social shrinkage in the city (Fig. 8c). On the space dimension, there were 27 cities that experienced an increase in shrinkage from 2005 to 2010 to 2010–2015, with the main shift in the direction of the bottom of shrinkage (including unshrinkage) to severe shrinkage in 16 cities, another nine cities where the degree of shrinkage did not change, and 20 cities where the degree of shrinkage weakened. From 2010 to 2015 to 2015–2020, the amount of cities with increasing degrees of shrinkage decreases. Although the number of cities with unchanged shrinkage has not decreased, the number of cities with severe shrinkage has increased by 5 and the number of cities with weakened shrinkage has decreased by 2 compared to the previous period. Overall, the degree of urban shrinkage in the YRB in the spatial dimension is increasingly serious. The possible reason for this is that some of the cities have problems of inefficient land utilization due to limited spatial resources in the process of urban development and unreasonable urban planning, etc., which in turn affects the spatial development of cities and triggers urban shrinkage (Fig. 8d). On the comprehensive dimension, there were 23 cities where the shrinkage worsened from 2005 to 2010 to 2010–2015, 8 cities where the shrinkage remained unchanged, and 17 cities where the shrinkage weakened. From 2010 to 2015 to 2015–2020, the number of cities with increased shrinkage decreased by three, the number of cities with unchanged shrinkage decreased by five, and the number of cities with decreased shrinkage increased by six. It shows that due to the transformation and upgrading of technological industries and the reform of institutional mechanisms in some cities, the competitiveness of enterprises and high-tech has been enhanced, so urban shrinkage has decreased (Fig. 8e).

### Characteristics of shrinking spatial cluster of cities in the YRB

**Fig. 9:**
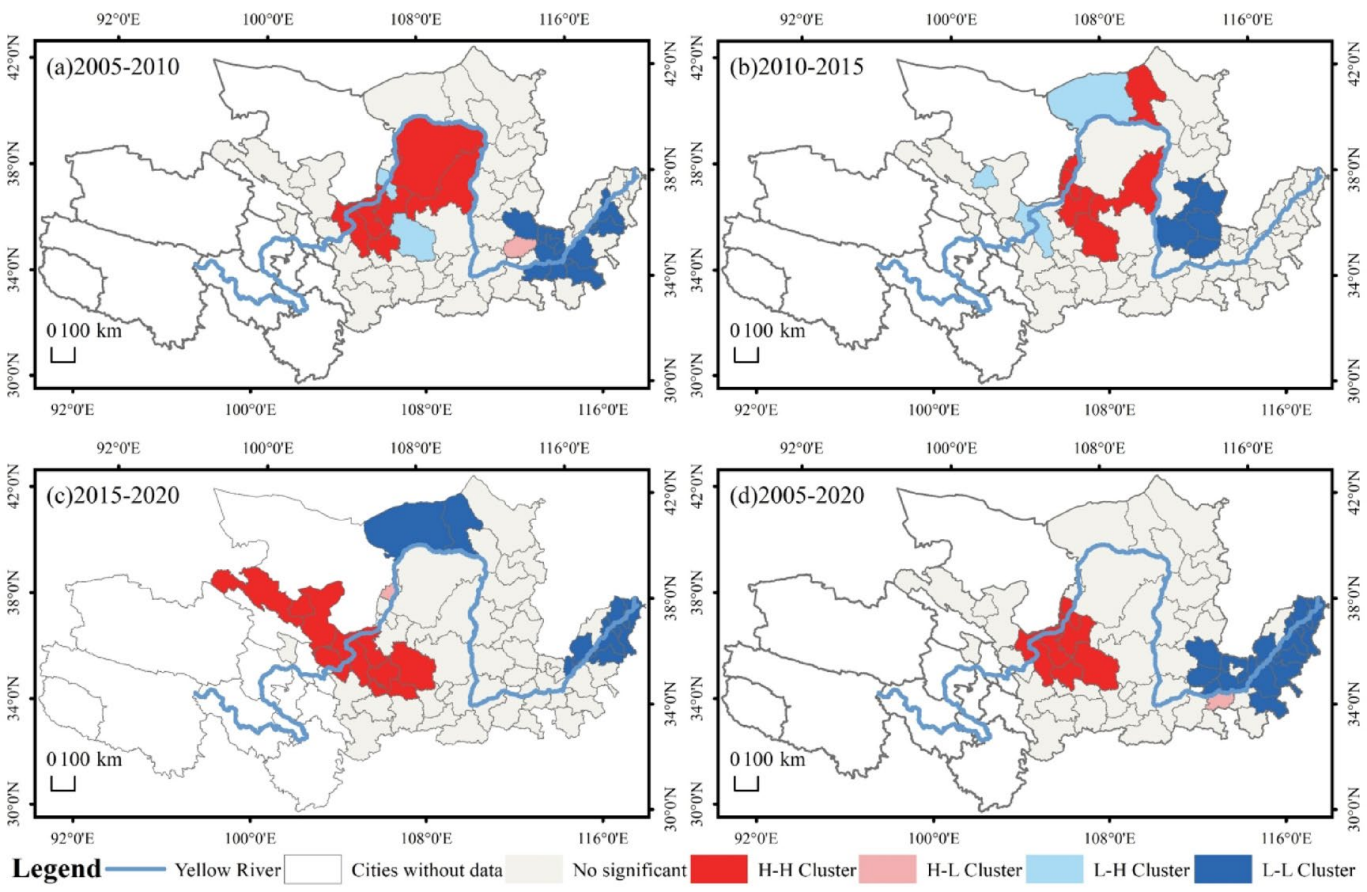
Characteristics of spatial agglomeration of shrinking cities in the YRB.

To fully reveal the spatial correlation of the contraction intensity of the cities in the YRB, this study analyzes the spatial agglomeration characteristics of urban shrinkage under each period. The results showed that Moran’s I was 0.388, 0.307, 0.454 and 0.467 for the four periods of 2005–2010, 2010–2015, 2015–2020 and 2005–2020, respectively, and all passed the 0.05 significance test. This indicates that the growth and shrinkage of cities within the YRB show a significant positive spatial correlation, which is further strengthened. Further local spatial autocorrelation calculations were performed to obtain the LISA map of shrinking cities in the YRB (Fig. 9). In 2005–2010, the regions with Ningxia Hui Autonomous Region, Ordos, Wuhai and Yulin as the core formed HH region, that is, the urban growth cluster area, while Qingyang formed HL region, that is, the shrinking and enveloping growth area (Fig. 9a). In 2010–2015, the HH zone was the upper region centered on the Ningxia Hui Autonomous Region and Qingyang and Yulin, the LH zone was the region centered on Jinchang, Baiyin, and Bayannur, and the LL zone was mainly located in the southern region of Shanxi Province centered on Jinzhong, Linfen, and Changzhi (Fig. 9b). In 2015–2020, the HH zone is mainly located in Gansu Province and Zhongwei and Guyuan areas, and the LL zone is primarily located in the lower reaches of the Yellow River as well as the areas centered on Bayannur and Baotou (Fig. 9c). In 2005–2020, a clear east-west bifurcation is shown, which means that the lower reaches and the southern part of Shanxi Province are the LL zone, which means shrinkage agglomeration, and the upper reaches, centered on Zhongwei, are the HH zone, which means urban growth agglomeration (Fig. 9d).

## Analysis of influencing factors

### Correlation analysis

Before the regression analysis, the existence of correlation between the influencing factors was tested. The Pearson correlation analysis of the intensity of urban shrinkage with each variable was conducted using SPSS.27 software, and the results are shown in Table 4. Most of the influence factors are extremely close to the city, except for the natural population growth rate, population density, the level of industry, service industry, and services accounted for the proportion of industry and service industry other 14 influence factors have passed the 0.05 significance test, in which the total population at the end of the year, the registered unemployed population in cities and towns, science accounted for the proportion of fiscal expenditures, the total value of industrial output above the large-scale industry, and the investment in fixed assets and so on, the 12 influence factors are all at the 0.01 level of significance. Therefore, it can be considered that there is a strong correlation between the selected influencing factors and urban shrinkage, so that the 14 evaluating indicators that passed the test can be used to analyze the influencing factors.

**Table 4:** Correlation test between each influence factor and shrinkage strength index.

Variable name	Variable	Correlation	Significance
Total population at year end	X_1_	− 0.399**	0.000
Natural population growth rate	X_2_	− 0.012	0.867
Population density	X_3_	− 0.404**	0.000
Urban registered unemployed population	X_4_	− 0.215**	0.003
Industrialization level	X_5_	− 0.105	0.155
Service level	X_6_	0.066	0.371
Science as a proportion of fiscal expenditure	X_7_	− 0.270**	0.000
Education as a proportion of fiscal expenditure	X_8_	− 0.063	0.394
Gross industrial output value above scale	X_9_	− 0.390**	0.000
investment in fixed assets	X_10_	− 0.379**	0.000
Greening coverage in built-up areas	X_11_	− 0.461**	0.000
PM2.5 concentration	X_12_	− 0.376**	0.000
Average altitude	X_13_	0.427**	0.000
Average slope	X_14_	0.164*	0.026
Terrain relief	X_15_	0.154*	0.035
Average annual temperature	X_16_	− 0.399**	0.000
Average annual rainfall	X_17_	− 0.370**	0.000
NDVI	X_18_	− 0.403**	0.000

**Significant correlation at the 0.01 level, *Significant correlation at the 0.05 level.

### Random forest regression validation

**Fig. 10:**
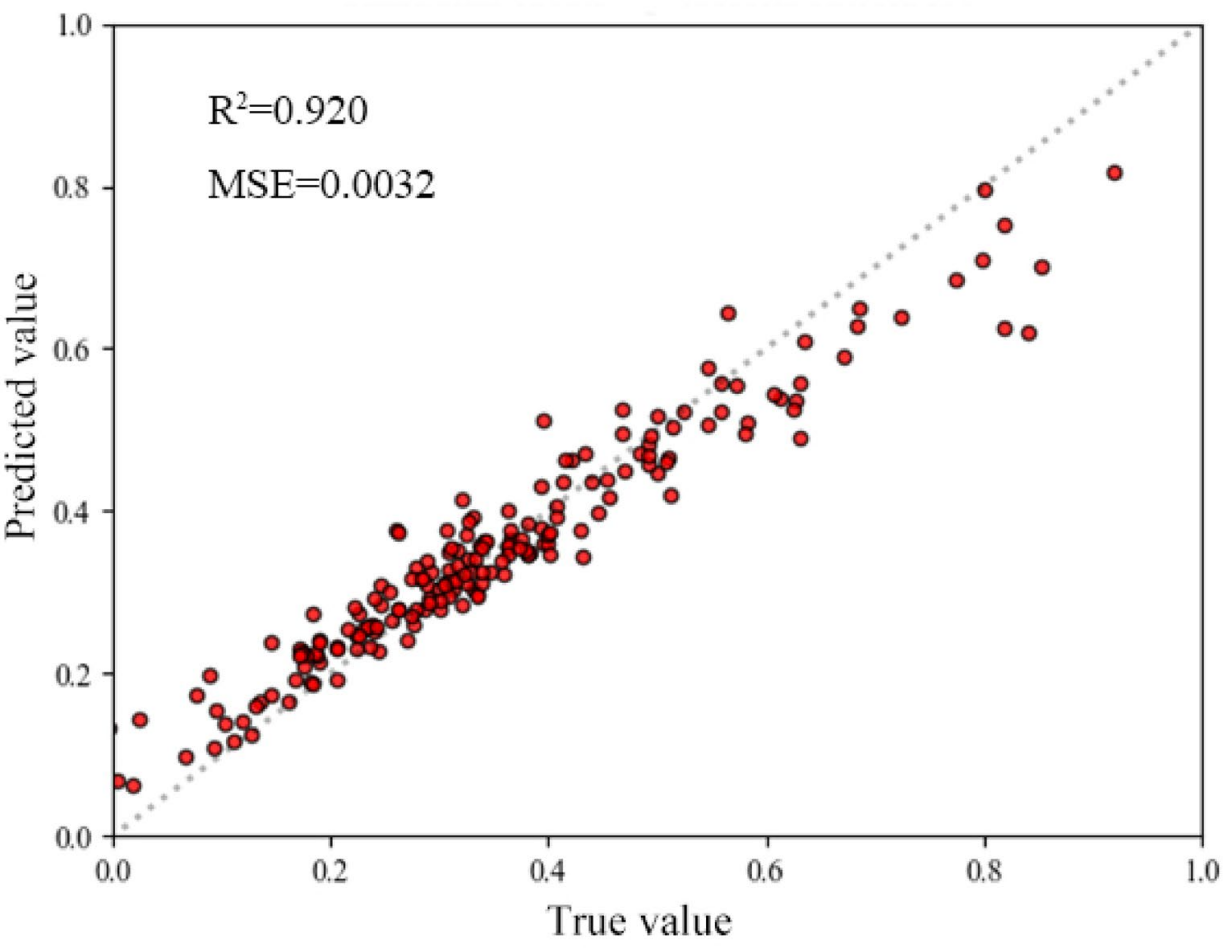
Random forest regression validation scatterplot.

The 14 influencing factors that passed the Pearson correlation test were entered into the random forest regression model, which was used to analyze the relationship between urban shrinkage intensity and each influencing factor, and to further evaluate its regression accuracy. In this study, the random forest regression was performed by calling sklearn module on Python platform, and after several debugging, the parameters n_estimators = 800, criterion = MSE were selected, and the rest of the parameters were defaulted. The fitted value (R^2^) and mean absolute error (MSE) of the random forest model were calculated and the corresponding scatter plots of fitted and predicted values were plotted (Fig. 10).

From the scatter plot of the random forest regression model, it can be seen that its R² is as high as 0.920 and the MSE is 0.0032.This indicates that the random forest regression model can fit the data better and its prediction results have smaller errors, with good precision and goodness of fit, and its results are trustworthy and suitable for the analysis of importance of influencing factors.

### Ranking and analyzing the importance of influencing factors

**Fig. 11:**
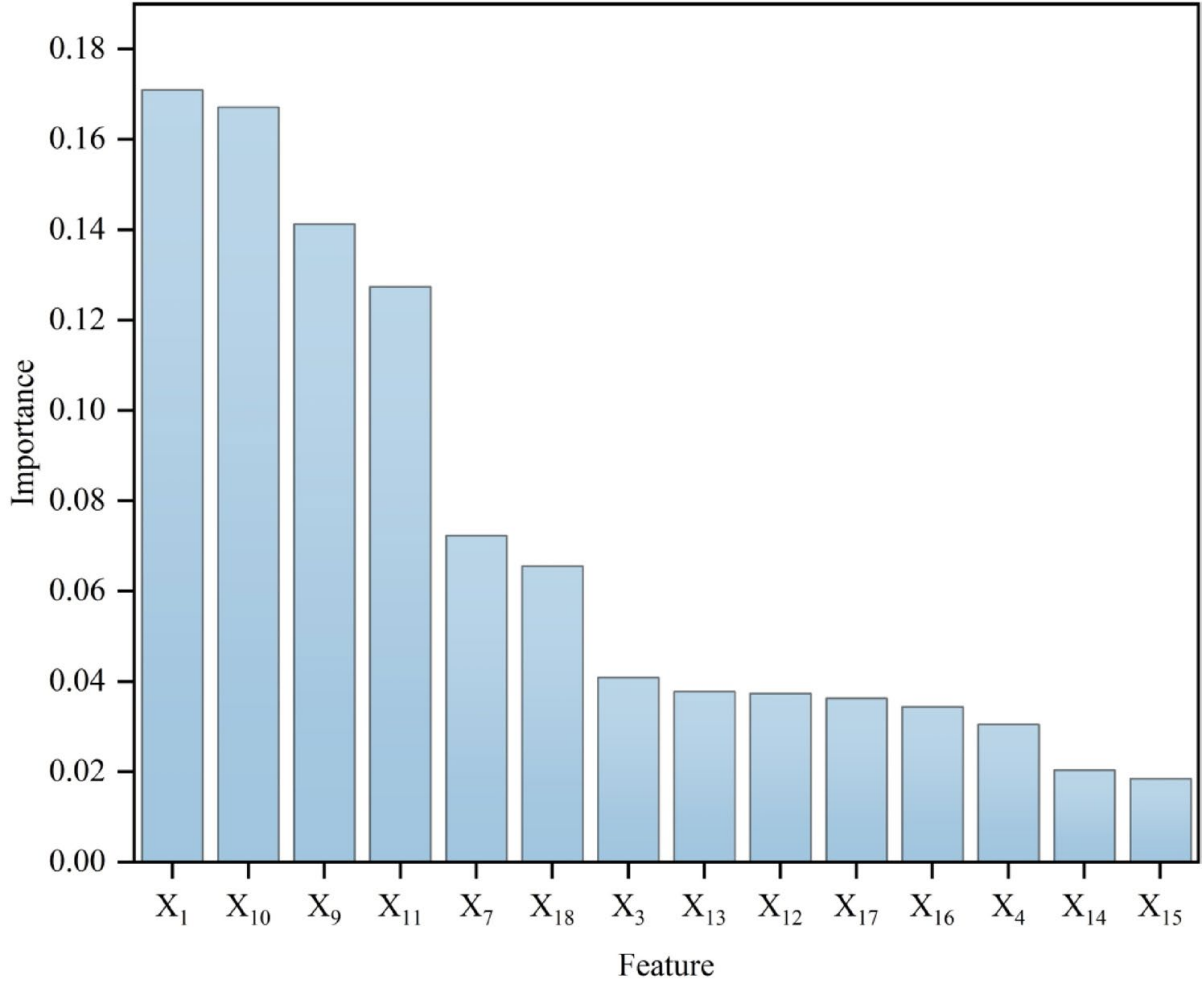
Ranking of importance of impact factors.

The IncMSE method was utilized to obtain the importance ranking of the influential factors under RF regression (Fig. 11). IncMSE ranking results show that the top 4 influencing factors are X_1_ (17.09%), X_10_ (16.71%), X_9_ (14.12%), X_11_ (12.74%), and the proportion of the top 4 influencing factors reaches 60.66%, which fully indicates that the top 4 influencing factors are the dominant factors for the occurrence of shrinkage in cities in the YRB, in addition, X_15_ is ranked 18th with an IncMSE of 1.84%, which indicates that the degree of terrain undulation has less influence on city shrinkage.

**Fig. 12:**
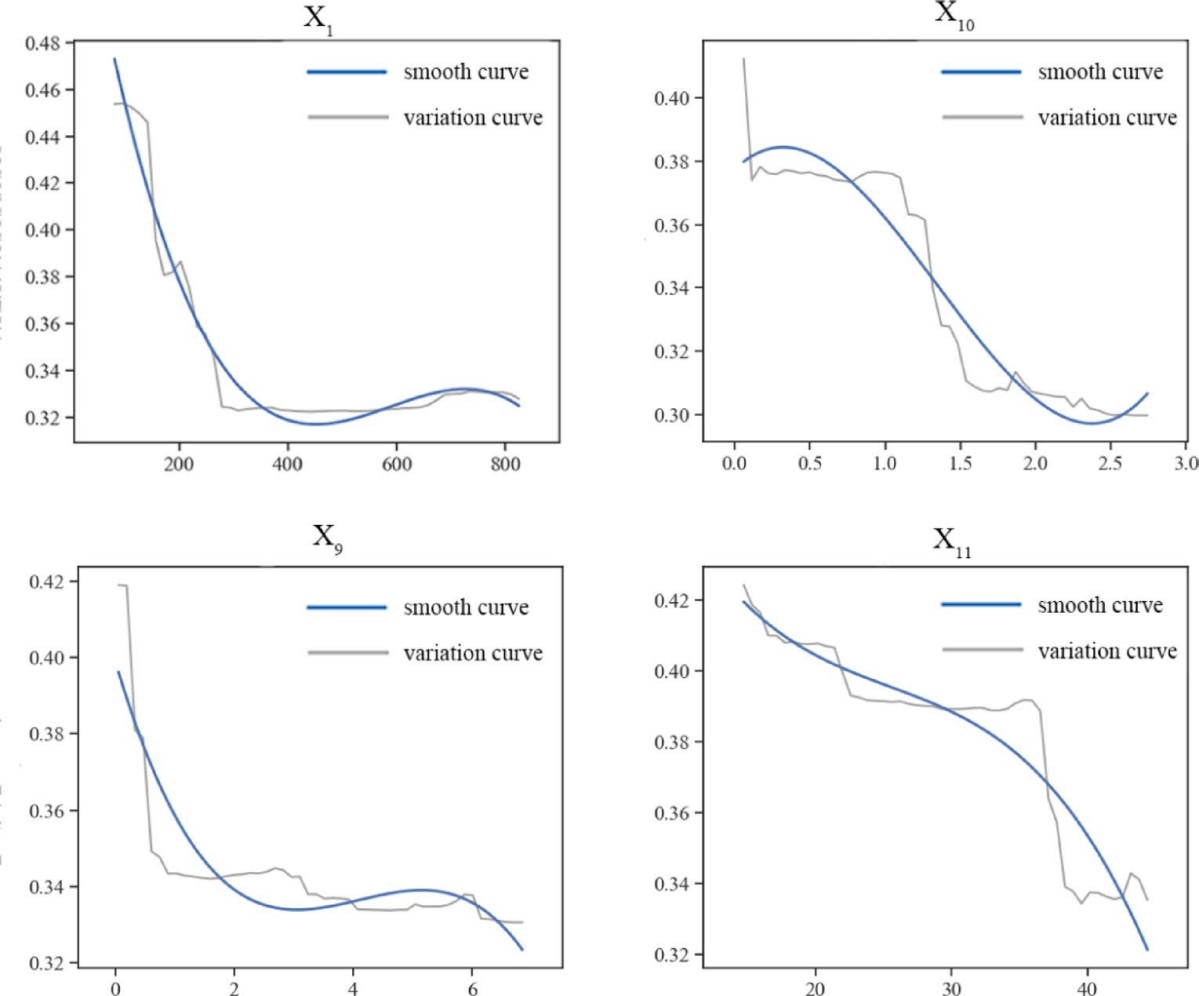
Partial dependence diagram of the top four influencing factors.

Based on the results of the importance of the influence factors, the obtained RF regression model was used to analyze further the influence of the top 4 influence factors on shrinking cities, and the influence factor partial dependence graph was plotted (the blue solid line is the smoothed curve; the grey solid line is the change curve). Based on the influence factors and urban growth and shrinkage of the bias-dependence smoothing curve can be seen (Fig. 12), the greatest impact of the total population at the end of the year (X_1_) on the impact of urban contraction shows a trend of increasing and then decreasing, which indicates that the total population of the city is less, which means that the impact of urban contraction of the other elements of development are also less urban contraction is more likely to occur. On the contrary, as the total population increases, other development factors are concentrated, the less likely the city shrinkage occurs. Fixed asset investment (X_10_) on the city’s shrinkage and growth trend shows a rising - falling - rising trend, the possible reasons: when the fixed asset investment is low will affect the city’s future development potential, too high fixed asset investment may be due to the limitations of the city’s size, the city to provide employment opportunities can not meet the demand for labor, or the pressure of competition for employment is too large, which leads to the labor force tend to leave the city to look for other opportunities. the city to look for other opportunities. The larger the total industrial output value above scale (X_9_), the greater the possibility of shrinkage, indicating that the current industrial structure of the cities in the YRB is still dominated by the secondary industry. The transformation of the industrial structure not only helps to enhance the economic vitality of the city, but also attracts more population inflow through the introduction of new industries and technological innovations, which in turn effectively curbs the trend of city shrinkage. By adjusting the industrial structure, cities can get rid of the dependence on traditional single industry, develop diversified and high value-added industries, create more employment opportunities, thus attracting the settlement of foreign population and the return of laborers, and driving the recovery and sustained growth of the city’s economy. Especially in some resource-oriented cities, industrial structure transformation is crucial to alleviate urban shrinkage. The trend of the green coverage ratio of built-up areas (X_11_) shows that the more severe the shrinkage, the higher the proportion of green coverage ratio of built-up areas in cities. The possible reason for this is that cities in underdeveloped regions do not have advantageous industries compared to other developed regions, and are unable to attract talents and capital investment, resulting in slow economic development. Such cities attract talent by improving the quality of the living environment, but the attraction is limited, which may trigger further aggravation of urban shrinkage.

## Discussion

### Urban shrinkage complexity

Revealing shrinking cities from a multi-faceted perspective offers a framework for delving into the intricacies of shrinking cities at the regional level. Most of the existing research has emphasized the identification of populations and economic dimensions. Liu et al.^46^ argue that in addition to the population and economic dimensions, which are considered to be the two most important dimensions, the social dimension is also essential because it is highly interactive with economic and population changes; He et al.^23^ argue that shrinkage affecting cities is caused by the spatial increase or decrease in the concentration and size of population and economic factors. Both agree that urban shrinkage is influenced by multiple dimensions, which is consistent with Hartt^72^. A multidimensional approach was also used to analyze urban shrinkage in this study, and cities experiencing population shrinkage are more similar to cities experiencing social shrinkage, suggesting that both population and social service functions may be affected by common causes. An increase in population can put pressure on health services, educational resources, and infrastructure. On the other hand, a decrease in population will lead to a mismatch between the development of social service functions and residents, and a decrease in other social resources such as social infrastructure, which will lead to the development of the city in a vicious circle, and the phenomenon of “the strong getting stronger, and the weak getting weaker” in the development of the city. From the above research results, it can be seen that the similarity between demographic and social dimensions reflects the complexity of urban development. Therefore, analyzing urban shrinkage from a multidimensional perspective may be a direction for future research on urban shrinkage. Empirical studies of multidimensional shrinkage in the study of urban shrinkage in the YRB are very limited, and are mainly analyzed from the population and economic dimensions^39,41^ as well as nighttime lighting data^40^. So it is necessary to analyze urban shrinkage in the YRB from population, economic, social and space dimensions. From the results of the influencing factors, this study, along with Tong et al.^52^ and Jin et al.^39^ concluded that the population element is the critical factor influencing urban shrinkage. Industrial transformation and upgrading play an essential role in urban shrinkage, consistent with the findings of Guo et al.^9^ and Zhang et al.^10^. In addition, the science as a proportion of fiscal expenditures is an essential factor in urban shrinkage, confirms the conclusions of Guan et al.^73^ on the negative impact of inadequate fiscal spending on the development of shrinking cities with shrinking populations. The ecological environment of the YRB is extremely fragile^38,74^ and with the over-exploitation of mineral and energy resources, most of the cities with a relatively homogeneous industrial structure are on the verge of resource depletion, thus triggering urban shrinkage.

### Urban shrinkage management adapted to local contexts

**Fig. 13:**
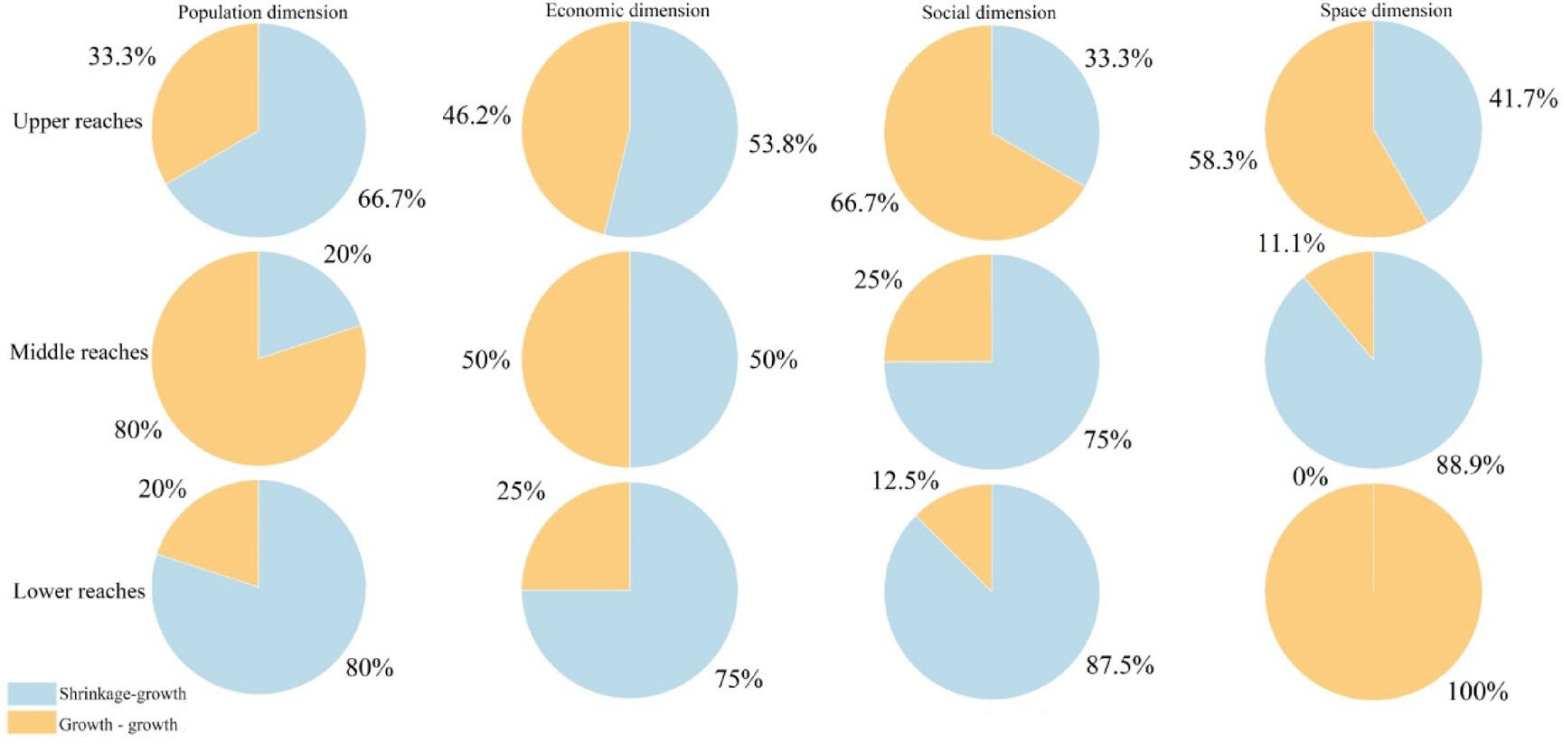
Ratio of urban shrinkage and growth in each dimension of the YRB.

The development pattern within a region is often composed of both contracting and growing cities, but scholars have primarily focused on how to increase the resilience of growing cities, while the resilience of contracting shrinking cities tends to be easy to ignore^75^. Eraydin and Özatagan^76^ summarize the policy agendas and governance practices adopted by shrinking cities from a resilience perspective to inform global urban shrinkage management. Previous studies have shown that it is necessary to formulate differentiated urban shrinkage management systems^46^ in watershed areas for different types of shrinking cities, but geographical location information is often easily ignored^39^. Therefore, this study presents the process of urban shrinkage and growth in different river sections of the YRB based on various dimensions (Fig. 13). Shrinking cities on the population dimension are most resilient in the Upper YRB, accounting for 66.7% of all shrinking cities that realized growth. On the social dimension, just 33.3% of shrinking cities realized growth, showing that shrinking cities with social growth are the least resilient to risk. In the space dimension, growing contracting cities had the highest growth rate of 88.9%, indicating that increasing contracting cities had the highest and most resilient growth rates in the middle of the region. The proportion of growth in shrinking cities that realized social growth was highest in the lower reaches of the area, suggesting that socially growing shrinking cities in the area are the most resilient. In addition, many shrinking cities have realized growth as their populations continue to grow, indicating that population growth contributes to the upper reaches of urban resilience (Fig. 13).

Similarly, existing studies report many examples of watershed-scale urban shrinkage, such as the Huaihe River Ecological Economic Belt shrinkage cities mainly in the middle and upper reaches. The middle and upper reaches are characterized by resource-based cities, employing a high-input, high-consumption, and high-emission production mode. The level of resource consumption and environmental pollution is more severe than in the lower reaches^77^. Therefore, when formulating shrinkage management strategies, it is necessary to consider the geographic location of the city in the watershed, as well as functional positioning and other factors, by the principle of adapting to the local conditions, the time, and the city, based on the enhancement of the resilience of the city, and to formulate a differentiated response strategy for urban shrinkage.

### Limitation and future directions

The evaluation system of urban shrinkage in the YRB contains four dimensions: population, economy, society and space, but due to the complexity of urban space development and the causes of urban shrinkage, the problem of urban shrinkage tends to affect shrinkage for different reasons depending on the geographical area. In the future, more relevant indicators can be considered for inclusion in the evaluation system of shrinking cities in the context of different regions, and more influential factors potentially affecting urban shrinkage can be explored. Future research could comparatively analyze the differences in the spatial-temporal distribution of urban shrinkage occurring at different sizes and scales and the differences in the main influencing factors. The current research scale only goes to the prefecture-level and lacks analysis at the smaller scale (county and district level and township and street level). In addition, this study did not separately analyze the influencing factors of upstream, midstream and downstream shrinking cities, which could be used as a direction for future research.

## Conclusion

Urban shrinkage is a serious threat to the sustainability of urban development in the YRB. Consequently, the study of urban shrinkage in the YRB is of great significance to the high-quality development of the YRB. This study takes 62 cities in the YRB for which data are available as the study area, constructs the evaluation index system from multiple dimensions, combines the transfer matrix to characterize the evolution of urban shrinkage patterns, and introduces RF regression to analyze the influencing factors of urban shrinkage in the YRB and their influencing roles. The results show that the development of cities in the YRB is still dominated by growth, but the risk of potential shrinkage should be prevented. Looking at all dimensions, the amount of shrinking cities has decreased, but the degree of shrinkage has gradually deepened. From the obtained ranking of the weights of each influencing factor, it can be found that the population factor is the critical factor that determines the shrinkage or not of the cities in the YRB.

From the results of this study, a multidimensional perspective helps to explore the spatial-temporal heterogeneity of urban shrinkage in the YRB. Implementing a multi-tiered urban management system will contribute to alleviating urban shrinkage in the YRB. Diversification and continuous upgrading of the city’s industrial structure effectively counteract urban shrinkage. Therefore, rational allocation of the industrial structure within the watershed, guiding the population to cluster in the lower reaches and industries to migrate to the upper reaches, as well as clarifying the developmental positioning and functions, can achieve efficient shrinkage management. In addition, there are more resource-oriented cities in the YRB, which should avoid a single industrial structure, leading to solidifying the development path and the resulting excessive resource and environmental costs. This study can provide a reference for shrinkage management and high-quality development of cities in the YRB.

## Data Availability

The authors declare that the data supporting the findings of this study are available within the paper, its supplementary information files.
